# Four Cases of Abnormal Neuropsychological Findings in Children with High Blood Methylmercury Concentrations

**DOI:** 10.1186/2052-4374-25-18

**Published:** 2013-09-24

**Authors:** Young-Seoub Hong, Dae-Seon Kim, Seung-Do Yu, Seong-Hwan Kim, Jong-Kuk Kim, Yu-Mi Kim, Jae-Ho Yu, Ji-Hyun Jung, Byoung-Gwon Kim

**Affiliations:** 1 Department of Preventive Medicine, College of Medicine, Dong-A University, Busan, South Korea; 2 Heavy Metal Exposure Environmental Health Center, Dong-A University, Busan, South Korea; 3 Department of Environmental Health Research, National Institute of Environmental Research(NIER), Incheon, South Korea; 4 Department of Psychiary, College of Medicine, Dong-A University, Busan, South Korea; 5 Department of Neurology, College of Medicine, Dong-A University, Busan, South Korea; 6 Department of Pediatrics, College of Medicine, Dong-A University, Busan, South Korea; 7 Department of Occupational and Environmental Medicine, Dong-A University Medical Center, Busan, South Korea

**Keywords:** Methylmercury, Neuropsychological findings, Fish consumption

## Abstract

**Background:**

Methylmercury (MeHg) easily crosses the blood–brain barrier and accumulates in the brain. Accumulated MeHg will cause neurological symptoms. We report four pediatric cases of neuropsychological findings with high blood MeHg concentrations.

**Case presentation:**

Four children were admitted for follow-up study because their total mercury (THg) concentration in the blood was found to be high during a national survey. Case 1 was a 9-year-old female with a 16.6 *μ*g/ℓ blood THg concentration in the survey. During admission, the blood THg, hair THg, and blood MeHg concentration(mercury indices) were 21.4 *μ*g/ℓ, 7.2 *μ*g/g, and 20.1 *μ*g/ℓ, respectively. In our neuropsychological examination, cognitive impairment and attention deficit were observed. Her diet included fish intake 2–3 times per week, and she had been diagnosed with epilepsy at 3 years of age. Case 2 was a 12-year-old male with blood THg of 15.4 *μ*g/ℓ in the survey and the mercury indices were 12.7 *μ*g/ℓ, 5.7 *μ*g/g, and 11.8 *μ*g/ℓ, respectively, on admission. He was also observed to have attention-deficit/hyperactivity disorder. Case 3 was a 10-year-old male child with blood THg of 17.4 *μ*g/ℓ in the survey, and the mercury indices on admission were 21.6 *μ*g/ℓ, 7.5 *μ*g/g and 21.5 *μ*g/ℓ, respectively. In his case, mild attention deficit was observed. Case 4 was a 9-year-old male with blood THg of 20.6 *μ*g/ℓ in the survey and the mercury indices were 18.9 *μ*g/ℓ, 8.3 *μ*g/g, and 14.4 *μ*g/ℓ, respectively, on admission. Mild attention difficulty was observed.

**Conclusion:**

We suggest that fish consumption may be the main source of MeHg exposure, and that MeHg may have been the cause of the neuropsychological deficits in these cases.

## Background

The toxicity of methylmercury (MeHg) in the human body has been demonstrated by historical incidents and large-scale epidemiological studies. Representative accidents of MeHg poisoning include Minamata disease in Japan [1] and the Iraqi mercury poisoning [2]. From these accidents, MeHg exposure has been shown to have fatal effects on the development of the fetal brain and nerves as well as on adults [3]. In addition, there have been a number of large-scale epidemiological studies on the health effects of MeHg including those in the Faroe Islands [4], Seychelles [5], and New Zealand [6]. The studies in the Faroe Islands and New Zealand reported that pregnant women’s exposure to MeHg through fish intake affected their children’s neuro-cognitive development.

The chronic health effects of mercury that have been reported in Korea are mostly occupational poisoning, along with a few cases of acute health problems caused by non-occupational factors [7]. The authors performed a thorough examination for four children whose were referred to follow-up study due to a mercury concentration in the body exceeding the international standard (15 *μ*g/ℓ, German HBM II) [8] in a community epidemiological survey of 1,097 students among grades 4 to 6 in 19 elementary schools of the Gyeongsang-do area who xparticipated in 2010 because the blood mercury concentration among adults living in certain areas of Gyeongsang-do was found to be very high during the Korea National Environmental Health Survey (KNEHS) in 2007 and neuro-psychological deficits that were believed to be induced by MeHg had been observed. We report here the cases with a literature review.

## Case presentation

### Case 1

Patient: A female, 9 years and 10 months old

Chief complaint: High blood total mercury (THg) concentration

Presenting illness: She visited our hospital in order to determine the presence of any health problems related to mercury exposure as her blood THg concentration was 16.6 *μ*g/ℓ in the Survey on Elementary School Children’s Exposure to Mercury and Health Effects in the Yeongnam Area and the Survey on the Effect of Abandoned Metal Mines on Residents’ Health conducted in July through October 2010 by the National Institute of Environmental Research (THg mean: 2.70 *μ*g/ℓ).

History: The patient had been diagnosed with epilepsy and was taking medication, and did not have any other medical history except allergic rhinitis. She had a very strong preference for fish and shellfish intake (particularly shark meat [*dombegi*]), eating fish almost every day. The parents both had hypertension but no other clinical history.

Family history: In the guardian (mother), who was tested for reference, the THg concentration in the blood was 8.48 *μ*g/ℓ, blood MeHg concentration 3.18 *μ*g/ℓ, and urinary mercury concentration in spot urine 6.33 *μ*g/ℓ.

Lab exam: The girl underwent a blood chemistry test on admission. The abnormal finding was increased triglycerides, and the other findings were within normal limits (Table 1).

**Table 1:** Laboratory findings and blood metal concentration of cases on admission

**Substance tested (Unit)**	**Case 1 (F/9Y)**	**Case 2 (M/12Y)**	**Case 3 (M/10Y)**	**Case 4 (M/9Y)**	**Ref.**
AST (IU/L)	19	20	24	34	10-35
ALT (IU/L)	13	16	16	21	0-35
BUN (mg/dL)	11	11	16	11	8-26
Glucose (mg/dL)	96	91	94	93	76-110
Total cholesterol (mg/dL)	188	243	195	198	130-250
Triglyceride (mg/dL)	269	337	241	77	30-200
HDL cholesterol (mg/dL)	47	45	51	61	M: 31–67
					F: 45-74
LDL cholesterol (mg/dL)	108	151	110	119	65-140
CK-MB (U/L)	14	18	10	16	0-15
LDH (IU/L)	342	393	361	595	120-520
Blood mercury (*μ*g/ℓ)	21.40	12.1	21.6	18.86	0-5
Blood methylmercury (*μ*g/ℓ)	20.1	11.8	21.5	14.4	0-5
Urinary mercury (*μ*g/ℓ)	1.4	2.785	2.42	1.77	0-5
Hair mercury (*μ*g/g)	7.2	5.7	7.5	8.3	0-1
Blood lead (*μ*g/㎗)	1.31	1.97	1.51	1.095	0-5
Blood cadmium (*μ*g/ℓ)	0.82	0.78	0.86	0.30	0-5
Ref: Blood mercury at survey (*μ*g/ℓ)	16.6	15.4	17.4	20.6	0-5

*AST* Aspartate Aminotransferase, *ALT* Alanine Aminotransferase.

*BUN* Blood urea nitrogen, *HDL* High-density Lipoprotein, *LDL* Low-density Lipoportein.

*CK-MB* Creatine Kinase, muscle-brain type, *LDH* Lactate Dehydrogenase.

Neuropsychological exam: As assessment tools, we used the Korean Wechsler Intelligence Scale for Children (K-WISC-III), House-Tree-Person Drawing Test (HTP), Kinetic Family Drawing Test (KFD), Rey-Kim Memory Test for children, Kim’s Frontal Executive Function Neuropsychological Test (K-FENT), Conners’ Abbreviated Parent Rating Scale (ACRS), Social Maturity Scale (SMS), and Child Behavior Checklist (CBCL). When general cognitive functioning was assessed with the K-WISC-III, the patient showed intellectual performance at a mild mental retardation level [general intelligence: 56, verbal intelligence: 49, performance intelligence: 71], and belonged to below the 1% tile equivalent to the 99^th^ out of 100. According to the results of the Rey-Kim Memory Test, the patient’s memory quotient (MQ: 53) and executive intelligence quotient (EIQ: 53) were both low enough to be considered disordered, showing a pattern similar to that of the patient’s IQ. In addition, the patient was reported to have as low a level as mental retardation in all of the areas of common sense, numerical calculation, vocabulary, and logical thinking, but her social maturity level was 9.0 (SQ 97.3), which was relatively higher than her current IQ. In addition, the results of other neurological tests were found to be within normal limits. From these results, it is believed that the patient had mild mental retardation and delayed language development accompanied by cognitive dysfunction and difficulty in both attention and concentration.

### Case 2

Patient: A male, 12 years and 7 months old

Chief complaint: High blood THg concentration

Presenting illness: The boy visited our hospital in order to be evaluated for the presence of any health problems related to mercury exposure, as his blood THg concentration was 15.4  *μ*g/ℓ in the same survey described above.

History: The patient did not have a medical history except for allergic rhinitis. He had a very high preference for fish and shellfish intake (specifically, dombegi), eating fish almost every day. Glaucoma in the right eyeball was suspected upon ophthalmologic examination.

Lab exam: The patient underwent a blood chemistry test on admission. The abnormal findings were increased triglycerides and total cholesterol. The other findings were within normal limits (Table 1).

Neuropsychological exam: When general cognitive functioning was assessed with the K-WISC-III, the patient showed intellectual performance at a boundary level [general intelligence: 77, verbal intelligence: 75, performance intelligence: 85], and belonged to the 6.3% tile, which was equivalent to the 94^th^ out of 100. He had a level as low as mild mental retardation in calculation, attention, and visual-motor coordination. Furthermore, his verbal intelligence was somewhat lower than his performance intelligence, and his performance was low in factors related to attention concentration. From the results of the Rey-Kim Memory Test, the patient’s MQ was 80, which was below the average and similar to the patient’s IQ. The results of the other neurological tests were within normal limits. These findings suggested with a diagnosis of attention deficit hyperactivity disorder (ADHD).

### Case 3

Patient: A male 10 years and 8 months old

Chief complaint: High blood THg concentration

Presenting illness: The patient visited our hospital in order to be evaluated for any health problems related to mercury exposure, as his blood The THg concentration had been 17.4 *μ*g/ℓ from the same survey.

History: The patient did not have any remarkable medical history. He had a very high preference for fish and shellfish (particularly dombegi) intake, but he had been restricting his fish intake since the community survey.

Family history: In the guardian (mother), who was tested as a reference, the THg concentration in the blood was 22.47  *μ*g/ℓ, blood MeHg concentration 21.3  *μ*g/ℓ, and urinary mercury concentration in spot urine 1.69  *μ*g/ℓ.

Lab exam: The boy underwent a blood chemistry test on admission. The abnormal finding was increased triglycerides, while the other findings were within normal limits (Table 1).

Neuropsychological exam: When general cognitive functioning was assessed with the K-WISC-III, the patient showed intellectual performance at an average level [general intelligence: 95, verbal intelligence: 96, performance intelligence: 95] and belonged to the 36.9% ile, equivalent to the 63^rd^ out of 100. According to the results of the Rey-Kim Memory Test, the patient’s MQ (95) and EIQ (99) were both at the average level. However, he was below the average in visual acuity, acquired common sense, design fluency, and visual imitation ability, and was even lower, at a boundary level, in visual immediate recall and delayed recall, attention concentration, and numerical calculation. Meanwhile, the results of the other neurological tests were within normal limits. These findings also suggested somewhat low attention concentration.

### Case 4

Patient: A male, 9 years and 6 months old

Chief complaint: High blood THg concentration

Presenting illness: The patient visited our hospital in order to be checked for the presence of any health problems related to mercury exposure, as his blood THg concentration was 20.6 *μ*g/ℓ from the same survey.

History: The patient had a very high preference for fish and shellfish (dombegi) intake, eating fish twice a week.

Family history: In the guardian (father), who was tested as a reference, the blood THg concentration was 22.68 *μ*g/ℓ, the blood MeHg concentration 20.0 *μ*g/ℓ, and the urinary mercury concentration in spot urine 4.51 *μ*g/ℓ.

Lab exam: The boy underwent a blood chemistry test on admission, and the results were within normal limits (Table 1).

Neuropsychological exam: When general cognitive functioning was assessed with the K-WISC-III, the patient showed below average intellectual performance [general intelligence: 82, verbal intelligence: 84, performance intelligence: 83], and belonged to the 11.5% ile, equivalent to the 89^th^ out of 100. From the results of the Rey-Kim Memory Test, the patient’s MQ was 114, higher than the average. According to the results of the K-CBCL, there was no scale that increased significantly, but the degree of attention deficit/hyperactivity (ACRS score: 17) exceeded the cutoff value (16), with scores of ‘Excessively high’ for ‘Emotional change’, and ‘Very high’ for ‘Hyperactivity’, ‘Impulsiveness’, ‘Short attention concentration’, ‘Easy distraction’, ‘Easy frustration’, ‘Easy crying’, and ‘Unpredictable behavior’. The results of other neurological tests were found to be within normal limits. These findings suggested mild disturbance of attention.

## Conclusions

MeHg, as organic mercury, is known to be harmful, particularly to brain tissue. In adults, MeHg that has passed through the blood brain barrier mainly induces the loss of cerebral visual cortical neurons and cerebellar neurons, but in fetuses and children at the stage of brain formation and development, it has been known to induce more extensive and fatal damage by disrupting the division of brain cells, the formation of microtubules, and the migration of neurons [9-11]. In this sense, the children in our cases are considered to be in the effective age group of MeHg damage.

In these cases, the hair THg concentration was 5.7-8.3 *μ*g/g, which exceeds 5 *μ*g/g, the safe concentration limit without neurological effects suggested by the Joint Expert Committee on Food Additives (WHO/JECFA), and the blood THg (MeHg) was 12.7-21.61(11.8-21.50) *μ*g/ℓ, which is higher than the international standard suggested by HBM II [8]. The result is higher than the mean blood THg concentration of 2.42±1.02 *μ*g/ℓ among Korean elementary school children reported by the National Institute of Environmental Research (2006) in Korea, the blood THg concentration of 0.444 *μ*g/ℓ reported by NHANES (2003–2006) in the U.S. [12], and 0.23 *μ*g/ℓ among 3 to 14-year-old children in Germany [13].

Moreover, severe symptoms induced by mercury in infants vary according to the perinatal mercury exposure concentration. That is, with increasing mercury concentration, there appeared symptoms such as variable heart rate, memory impairment, visuospatial dysfunction, motor neuron disorder, speech disorder, delayed development, loss of vision and hearing, epileptic seizure, ataxia and cerebral palsy, and mental retardation [14-16]. In our cases, it was difficult to measure the level of perinatal mercury exposure, but based on available mercury exposure indices, abnormal neuropsychological findings observed in the young patients are believed to correspond to the spectrum of various symptoms as listed above.

Case 1 had epilepsy at the time of presentation. In previous studies, convulsive seizure caused by mercury poisoning has been reported, and Park *et al.*[17] in Korea described a case of death by mercury poisoning, in which a 15-year-old boy showed epileptic seizure before his death. Considering the common effects of mercury on the central nervous system, though the form of mercury exposure varies, it is possible to identify the neurological impact of mercury exposure.

MeHg, which occupies most of the blood THg concentration, as it did in our cases, can accumulate through the intake of fish and shellfish. Many studies in Korea and overseas have found evidence for the association between fish intake and mercury exposure. In our cases as well, the patients’ high blood mercury concentration can be explained by their intake of fish [18], in particular, shark meat (dombegi), which is known to have high mercury content [19].

Some countries are blocking the routes of mercury intake by regulating the consumption of fish with high mercury content. For example, the British government recommends that pregnant women, reproductive-aged women, and 16-year-old or younger children not eat swordfish because of its high mercury content. In Korea, however, there is no established regulation on fish intake for vulnerable groups, such as pregnant women and children. Thus, this case report is considered important for the management of vulnerable groups and communication of the risk of mercury exposure. In this situation, moreover, there may be many vulnerable groups exposed to mercury, including pregnant women and children, so we need more extensive and diverse analyses and epidemiological surveys based on these cases. Also like these cases, occupational and environmental medicine clinics should unearth more cases of disease resulting from heavy metal exposure. In conclusion, the authors examined four children who visited our institution for thorough examination due to a high blood mercury concentration. We report here the neuropsychological abnormal findings we observed, including mild mental retardation, ADHD, and low attention concentration. Based on this case report, efforts should be made to conduct expanded research as well as to reduce children’s exposure to mercury and prevent related health problems.

## Consent

Written informed consent was obtained from the patient’s guardian/parent/next of kin for the publication of this report and any accompanying images.

## Competing interests

The authors declare that they have no competing interests.

## Authors’ contributions

HYS and KDS conceived and designed the study. KJK, YJH and JJH were involved in acquisition of data. KSH and KYM performed the analysis and interpretation of data. HYS, KDS and KBG were involved in writing the manuscript. KBG and YSD performed the revision the manuscript critically for important intellectual content. All authors read and approved the final manuscript.

## References

[B1] Harada Y Miyamoto Y Nonaka I Ohta S Ninomiya T Electroencephalographic studies of Minamata disease in children Dev Med Child Neurol 1968 25 2 257 258 5653106

[B2] Cox C Clarkson TW Marsh DO Amin-Zaki L Tikriti S Myers GG Dose–response analysis of infants prenatally exposed to methyl mercury: an application of a single compartment model to single-strand hair analysis Environ Res 1989 25 2 318 332 10.1016/S0013-9351(89)80075-1 2473897

[B3] WHO Guidence for identifying populations at risk from mercury exposure 2008 Geneva: WHO

[B4] Grandjean P Weihe P White RF Debes F Araki S Yokoyama K Murata K Sorensen N Dahl R Jorgensen PJ Cognitive deficit in 7-year-old children with prenatal exposure to methylmercury Neurotoxicol Teratol 1997 25 6 417 428 10.1016/S0892-0362(97)00097-4 9392777

[B5] Myers GJ Davidson PW Shamlaye CF Axtell CD Cernichiari E Choisy O Choi A Cox C Clarkson TW Effects of prenatal methylmercury exposure from a high fish diet on developmental milestones in the Seychelles Child Development Study Neurotoxicology 1997 25 3 819 829 9339828

[B6] Crump KS Kjellstrom T Shipp AM Silvers A Stewart A Influence of prenatal mercury exposure upon scholastic and psychological test performance: benchmark analysis of a New Zealand cohort Risk Anal 1998 25 6 701 713 9972579 10.1023/b:rian.0000005917.52151.e6

[B7] Seo HJ Myung YS Jung HJ Kim MJ Seo YR Park KA Park CS Park JS Park JS Case reports : a case of acute lung injury and acute generalized exanthematous pustulosis induced by inhalation of mercury vapor Korean J Asthma Allergy Clin Immunol 2012 25 4 259 263

[B8] Becker K Kaus S Krause C Lepom P Schulz C Seiwert M Seifert B German Environmental Survey 1998 (GerES III): environmental pollutants in blood of the German population Int J Hyg Environ Health 2002 25 4 297 308 10.1078/1438-4639-00155 12068749

[B9] Hughes WL A physicochemical rationale for the biological activity of mercury and its compounds Ann N Y Acad Sci 1957 25 5 454 460 10.1111/j.1749-6632.1956.tb36650.x 13411943

[B10] Castoldi AF Coccini T Ceccatelli S Manzo L Neurotoxicity and molecular effects of methylmercury Brain Res Bull 2001 25 2 197 203 10.1016/S0361-9230(01)00458-0 11470315

[B11] Clarkson TW Mercury: major issues in environmental health Environ Health Perspect 1993 25 31 38 8354179 10.1289/ehp.9310031 PMC1519577

[B12] Caldwell KL Mortensen ME Jones RL Caudill SP Osterloh JD Total blood mercury concentrations in the U.S. population: 1999–2006 Int J Hyg Environ Health 2009 25 6 588 598 10.1016/j.ijheh.2009.04.004 19481974

[B13] Schulz C Angerer J Ewers U Heudorf U Wilhelm M Human Biomonitoring Commission of the German Federal Environment Agency Revised and new reference values for environmental pollutants in urine or blood of children in Germany derived from the German environmental survey on children 2003–2006 (GerES IV) Int J Hyg Environ Health 2009 25 6 637 647 10.1016/j.ijheh.2009.05.003 19589725

[B14] Klaassen CD Casarett and Doull’s Toxicology - The Basic Science of Poisons 1996 5 Blacklick, Ohio, U.S.A: McGraw-Hill

[B15] Grandjean P Murata K Budtz-Jorgensen E Weihe P Cardiac autonomic activity in methylmercury neurotoxicity: 14-year follow-up of a Faroese birth cohort J Pediatr 2004 25 2 169 176 10.1016/j.jpeds.2003.10.058 14760255

[B16] Thurston SW Bovet P Myers GJ Davidson PW Georger LA Shamlaye C Clarkson TW Does prenatal methylmercury exposure from fish consumption affect blood pressure in childhood? Neurotoxicology 2007 25 5 924 930 10.1016/j.neuro.2007.06.002 17659343 PMC2104472

[B17] Park HS Lim HS Huh BY Hahn HG Hwang YS Moon HR Hong KE A case report of a fatal mercury poisoning J Korean Acad Fam Med 1991 25 5 66 71 (Korean)

[B18] Kim CW Kim YW Chae CH Son JS Park SH Koh JC Kim DS The effects of the frequency of fish consumption on the blood mercury levels in Koreans Korean J Occup Environ Med 2010 25 2 114 121 (Korean)

[B19] SaKon J Health effects of mercury exposure through fish Yeungnam Univ J Med 2011 25 2 105 115 (Korean)

